# Water molecule switching heterogeneous proton-coupled electron transfer pathway†

**DOI:** 10.1039/d2sc07038c

**Published:** 2023-04-03

**Authors:** Zhonghuan Liu, Wei Peng, Yuhan Lin, Xinyu Lin, Shikang Yin, Shuhan Jia, Dongge Ma, Yan Yan, Peng Zhou, Wanhong Ma, Jincai Zhao

**Affiliations:** a Key Laboratory of Photochemistry, Beijing National Laboratory for Molecular Sciences, Institute of Chemistry, Chinese Academy of Sciences Beijing 100190 PR China whma@iccas.ac.cn; b Institute of Green Chemistry and Chemical Technology, School of Chemistry and Chemical Engineering, Jiangsu University Zhenjiang 212013 PR China dgy5212004@163.com; c Department of Electrical Engineering and Computer Science, University of Michigan Ann Arbor MI 48109 USA dpzhou@umich.edu; d Department of Chemistry, College of Chemistry and Materials Engineering, Beijing Technology and Business University Beijing 100048 China; e University of Chinese Academy of Sciences Beijing 100049 China

## Abstract

Figuring out the specific pathway of semiconductor-mediated proton-coupled electron transfer (PCET) driven by light is essential to solar energy conversion systems. In this work, we reveal that the amount of adsorbed water molecules determines the photo-induced PCET pathway on the TiO_2_ surface through systematic kinetic solvent isotope effect (KSIE) experiments. At low water content (<1.7 wt%), the photo-induced single-proton/single-electron transfer on TiO_2_ nanoparticles follows a stepwise PT/ET pathway with the formation of high-energy H^+^/D^+^–O=C or H^+^/D^+^–O–C intermediates, resulting in an inverse KSIE (H/D) ∼0.5 with ^*t*^Bu_3_ArO· and KSIE (H/D) ∼1 with TEMPO in methanol-*d*_0_/*d*_4_ systems. However, at high water content (>2 wt%), the PCET reaction follows a concerted pathway with a lower energy barrier, leading to normal KSIEs (H/D) ≥ 2 with both reagents. *In situ* ATR-FTIR observation and DFT calculations suggest that water molecules' existence significantly lowers the proton/electron transfer energy barrier, which coincides with our experimental observations.

## Abstract 2

Water-participation matters: the photo-induced single-proton/single-electron transfer pathway on TiO_2_ nanoparticles can be significantly switched from stepwise PT/ET to one-step CPET by adsorbed water molecules.

## Graphical abstract

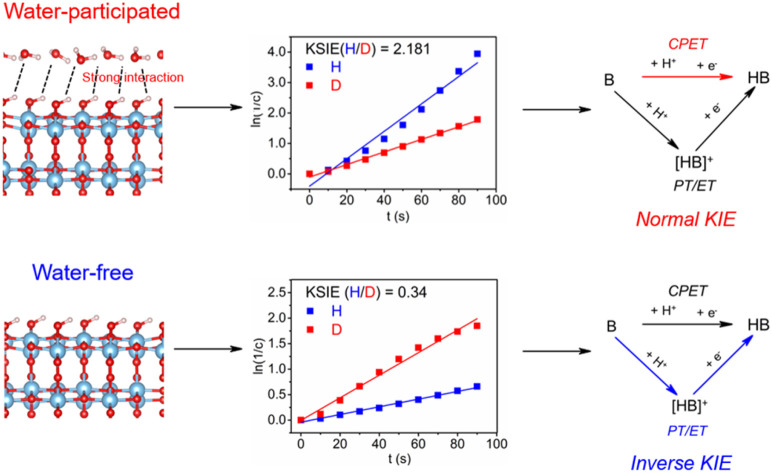

## Introduction

Proton-coupled electron transfer (PCET) reactions are pervasive in natural energy transfer processes, including photosynthesis, nitrogen fixation, and respiration.^1–4^ Metal oxide semiconductor (*e.g.*, ZnO, TiO_2,_ and SnO_2_) nanoparticle-mediated photo-induced electron transfer has been directly proven to be a proton-coupled process.^5^ Figuring out the internal relation between specific intermediate reaction pathways and the interfacial chemical environment is critical to the profound understanding and future design of targeted semiconductor nanoparticle-based photocatalytic systems with high efficiency.

Typically, under continuous light irradiation, metastable photo-induced electrons can be generated and constantly trapped on oxide semiconductor (*e.g.*, TiO_2_) nanoparticles in small-molecule hole-scavengers (*e.g.*, H_2_O, amines, or alcohols).^6,7^ Stabilized by protons (H^+^), photo-induced electrons (e_cb_^−^) on TiO_2_ nanoparticles were proved to be trapped at the outer surface as Ti_4c_^3+^ species,^8^ which can be consumed *in situ* or *ex situ* as reactive reducing reagents to accomplish many valuable and challenging chemical reactions without extra high-temperature and high-pressure conditions.^9–11^ The corresponding intermediate reaction pathway directly determines such a liquid/solid heterogeneous PCET process driven by photo-excitation. Compared to the stepwise electron-transfer followed by the proton-transfer (ET/PT) pathway and *vice versa* (PT/ET), concerted gain or loss of coupled e_cb_^−^ and H^+^ (CPET) typically favors redox reactions. The advantage of such a reaction pathway is that it avoids high-energy intermediates caused by localized charge accumulation,^12–14^ which makes it consistently exhibit a lower reaction energy barrier.^13^ Generally, the PCET pathway selectivity was long considered only determined by the proton concentration and specific activation path built by the reactive sites in reaction systems.^15^ Previous observations have revealed that the reduction potential of e_cb_^−^ varies sharply with the proton concentration near TiO_2_ electrodes, which exhibit different reactivities.^16^ Our previous work further distinguished the binding energy difference that reflects energy levels of electron trapping states on TiO_2_ nanoparticles between proton (1.3 eV) and proton-free (1.8 eV) systems by synchrotron radiation UV photoelectron spectroscopy.^17^ Furthermore, hybrid functional periodic density functional theory (DFT) calculations elucidate the impact of proton-coupled defects on the bond dissociation-free energies (BDFEs) of O–H bonds on anatase TiO_2_ surfaces, which are directly related to interfacial PCET thermochemistry.^18^ In dye-sensitized solar cells, the PCET model has been applied in monitoring geometric parameters, excitation, and electronic structures of free and Ti^4+^-bound squaraine dye solar cells.^19^ However, in a proton-excessive PCET system, *e.g.*, the most common solar energy conversion path of H_2_O → H_2_ or CO_2_ + H_2_O → C_*x*_H_*y*_O_*z*_ involving water participation, the impact of the aqueous interfacial chemical environment on the photo-induced PCET reaction pathway on semiconductor nanoparticles remains unclear. For instance, in some photo-reduction scenarios (*e.g.*, dehalogenation of aromatic halides, H_2_ production, and CO_2_ reduction), reaction efficiencies on semiconductor nanoparticle catalysts in H_2_O/organic mixtures were reported to be much higher than those in the pure organic phase.^20–22^ Although the effect of water as a direct reactant on photocatalytic performances has been reported in many terms, we still lack a consensual in-depth understanding of the cause of such phenomena. Therefore, determining whether these water-enhanced performances were directly achieved by regulating the PCET pathway is of utmost importance.

## Results and discussion

**Scheme 1:**
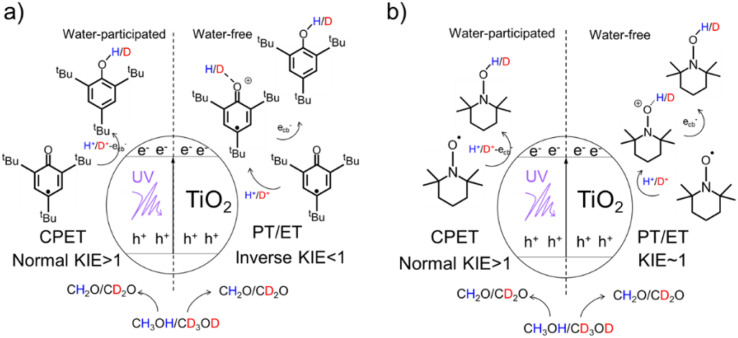
Mechanisms of TiO_2_ photocatalysis: a well-established CPET pathway induced free-radical reaction for (a) ^*t*^Bu_3_ArO· and (b)TEMPO substrates (left) and PTET pathway induced free-radical reaction (right).

Herein, by using a 2,4,6-tri-*tert*-butylphenoxyl radical (^*t*^Bu_3_ArO·) and 2,2,6,6-tetramethyl-piperidin-1-yl-oxyl (TEMPO) as single-electron/single-proton reagents and isotopically labeled methanol-*d*_0_/*d*_4_ as both the electron/proton donor and solvent, a series of delicate kinetic solvent isotope effect (KSIE (H/D)) experiments were carried out on TiO_2_ nanoparticles as catalysts (Scheme 1). We found an unexpected switch of the photo-induced PCET pathway from the stepwise PT/ET to CPET manipulated by a trace amount of adsorbed water molecules on TiO_2_ nanoparticles. *In situ* attenuated total reflection Fourier transform infrared spectroscopy (ATR-FTIR) observations confirmed a strong interaction between water molecules and photo-induced surface protons (mainly from the oxidation of methanol solvent) in the presence of e_cb_^−^. Such an interreaction directly affects the bond-dissociation of these protons from the TiO_2_ surface and favors proton-coupled reduction *via* a CPET pathway. Density functional theory (DFT) calculations were further employed to investigate the impact of water molecules on the proton bond-dissociation and electron transfer on the TiO_2_ surface.

### Determining the PCET pathway by KSIE (H/D) measurements

We first performed the KSIE (H/D) experiments on TiO_2_ nanoparticles under UV with the well-reported single-electron/single-proton radical reduction of ^*t*^Bu_3_ArO· (^*t*^Bu_3_ArO· + H^+^ + e_cb_^−^ → ^*t*^Bu_3_ArOH) and TEMPO (TEMPO + H^+^ + e_cb_^−^ → TEMPOH)^15,23^ by using isotopically labeled methanol-*d*_0_/*d*_4_ as the H/D solvent and electron/proton donor (CH_3_OH + h_vb_^+^ → CH_2_O + H^+^). ^1^H NMR spectra (Figure S1†) and mass spectra (Figure S2†) confirm no other major products except for the reduction product. All measurements were carried out under anaerobic conditions.

The KSIE (H/D) was determined using concentration-time profiles of ^*t*^Bu_3_ArO·/TEMPO that were *in situ* monitored in methanol-*d*_0_/*d*_4_ (H/D systems) by electron spin resonance (ESR) spectroscopy (for details of the KSIE experiments, see the ESI†). Control experiments were performed without TiO_2_ nanoparticles, confirming no reaction without a catalyst (Figure S3†). The commonly used commercial TiO_2_ nanoparticles (anatase-phase, without further treatment) were employed as a typical semiconductor nanoparticle catalyst. A direct normal KSIE (H/D) of 2.18 was observed with ^*t*^Bu_3_ArO· between methanol-*d*_0_/*d*_4_ systems (Figure S4†), suggesting a one-step CPET reaction pathway on pristine TiO_2_ nanoparticles.^24^ Moreover, with TEMPO as the reagent, a normal KSIE (H/D) of 3.73 was observed (Figure S5†). Our observation is consistent with the previously reported result by the Mayer group^5^ that the single-electron/single-proton transfer induced by photo-generated H^+^/e_cb_^−^ on TiO_2_ and ZnO nanoparticles follows a concerted pathway. However, when freshly high-temperature (773 K) calcined TiO_2_ nanoparticles were used as catalysts under otherwise identical conditions, the slower reaction kinetics of ^*t*^Bu_3_ArO· in methanol-*d*_0_ compared to methanol-*d*_4_ were observed with an unexpected inverse KSIE (H/D) of 0.34 (Figure S6†), indicating that the PCET reaction pathway was significantly changed. Noticeably, only when atomic hybrid modes adjacent to –H/D coordination were changed from sp^2^ to sp^3^, the subsequent bond-dissociation kinetic isotope effect (KIE) would appear with an inverse value, indicating the formation of a certain protonation intermediate configuration. Moreover, with TEMPO as the reagent, the KSIE (H/D) on calcined TiO_2_ nanoparticles was approaching ∼1 (Figure S7†) because the transformation of TEMPO into TEMPOH does not involve the hybridization change from sp^2^ to sp^3^. Control experiments have been performed with TiO_2_ nanoparticles calcined in an air, oxygen, or argon atmosphere. An inverse KSIE (H/D) ≤ 0.5 with ^*t*^Bu_3_ArO· and KSIE (H/D) ∼1 with TEMPO were always observed on calcined TiO_2_ nanoparticles regardless of atmosphere (Figures S8 and S9†). These results indicate that other effects from calcination rather than defect vacancies determine the switch of the KSIE (H/D) and corresponding PCET pathways.

The XRD result shows that the crystalline structure of anatase TiO_2_ nanoparticles was not changed after calcination and the crystallite diameter did not severely change (Figure S10 and Table S1†), which further excludes the impact of rutile formation as the major effect. We further compared the sedimentation behavior of a pristine/calcined TiO_2_ (2.5 g L^−1^) suspension by *in situ* UV-vis optical-fiber spectroscopy. As shown in Figure S11,† the light transmittance hardly changed within 90 s in both systems, suggesting that sedimentation is not the major effect for the KIE in our system.

**Fig. 1:**
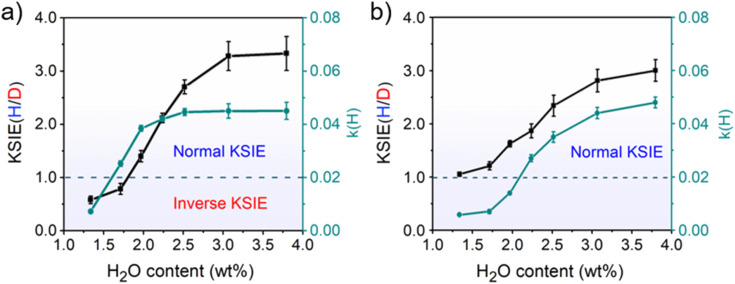
Profiles of the KSIE (H/D) and reaction rate parameter *k*(H) as functions of the water content in TiO_2_ nanoparticles with (a) ^*t*^Bu_3_ArO· and (b) TEMPO as the single-proton/single-electron acceptors under UV irradiation. Error bars were obtained by repeating the experiment three times under identical conditions.

The decreased KSIE value caused by calcination would recover to the normal KSIE value after the calcined TiO_2_ catalyst is stored in the air for one week (Figures S12 and S13†). We argue that the loss of trace water on TiO_2_ nanoparticles after calcination is the determining factor for the KSIE <1. Through the *in situ* diffuse reflection infrared Fourier transform spectroscopy (DRIFTS) characterization of TiO_2_ nanoparticles during calcination (298–773 K), we found that the featured signals of adsorbed water molecules (O–H stretching vibration band at 3200 cm^−1^ and H–O–H bending vibration at 1642 cm^−1^) severely decreased until they wholly disappeared with the increasing temperature. When the temperature exceeded 773 K, the loss of dissociated water (O–H stretching vibration band at 3694/3459 cm^−1^ without 1642 cm^−1^ bending vibration) was observed (Figure S14a†). However, with the temperature further returned to room temperature, although the ambient humidity can recover a certain amount of water adsorption on TiO_2_ nanoparticles, it would never be able to reach the pre-calcination level (Figure S14b†), demonstrating the inevitable loss of water content in TiO_2_ nanoparticles after high-temperature calcination. Moreover, by using thermogravimetric analysis (TGA) measurements, we found that the water content in pristine TiO_2_ nanoparticles long stored in the air was ∼3.76 wt% before calcination. In contrast, after 773 K calcination and returning to room temperature, the water content of the freshly calcined TiO_2_ nanoparticles was significantly reduced to ∼1.33 wt% (Figure S15†). To directly demonstrate the relationship between the water content of the catalyst and the KSIE (H/D) of photo-induced PCET reactions, we prepared a series of TiO_2_ nanoparticle catalysts with well-controlled water contents (1.33–3.80 wt%) by treating dried TiO_2_ nanoparticles in airflows with different humidities (Figure S16†) (for preparation methods see the ESI†). The coverage of water layers was calculated according to the surface Ti atoms on TiO_2_ nanoparticles (in terms of a perfect anatase {101} surface) and is summarized in Table S2,† corresponding to an increasing water coverage from ∼ one layer to ∼ three layers. With the increasing water content of TiO_2_ nanoparticles, the KSIE (H/D) with ^*t*^Bu_3_ArO· significantly increased from an inverse value of 0.34 to a normal value of 3.33 (Fig. 1a). In contrast with the KSIE(H/D) with ^*t*^Bu_3_ArO·, the KSIE (H/D) with TEMPO increased from a recessive value of ∼1 to a normal value of 3 (Fig. 1b), which provides solid evidence that the KSIE (H/D) value was directly determined using the water content in the TiO_2_ nanoparticle catalyst. Reaction rate parameters in methanol (*k*(H)) with both radical reagents show a similar trend that increases with the increasing water content (Fig. 1a and b), which is consistent with the water-determined KSIE (H/D). We further employed a well-reported Fe(iii)-1,10-phenanthroline spectrometric titration method (for details of the titration method see the ESI†) to quantify the oxidation of CH_3_OH/CD_3_OD on different TiO_2_ samples.^26^ As shown in Figure S17.† KIEs for methanol-d_0_/*d*_4_ oxidation on both dry (water content∼1.3 wt%) and wet (water content ∼ 3.8 wt%) TiO_2_ are identical at ∼1.3, indicating that the changed KIE value with increasing water content in the TiO_2_ catalyst is not from the methanol oxidation.

**Scheme 2:**
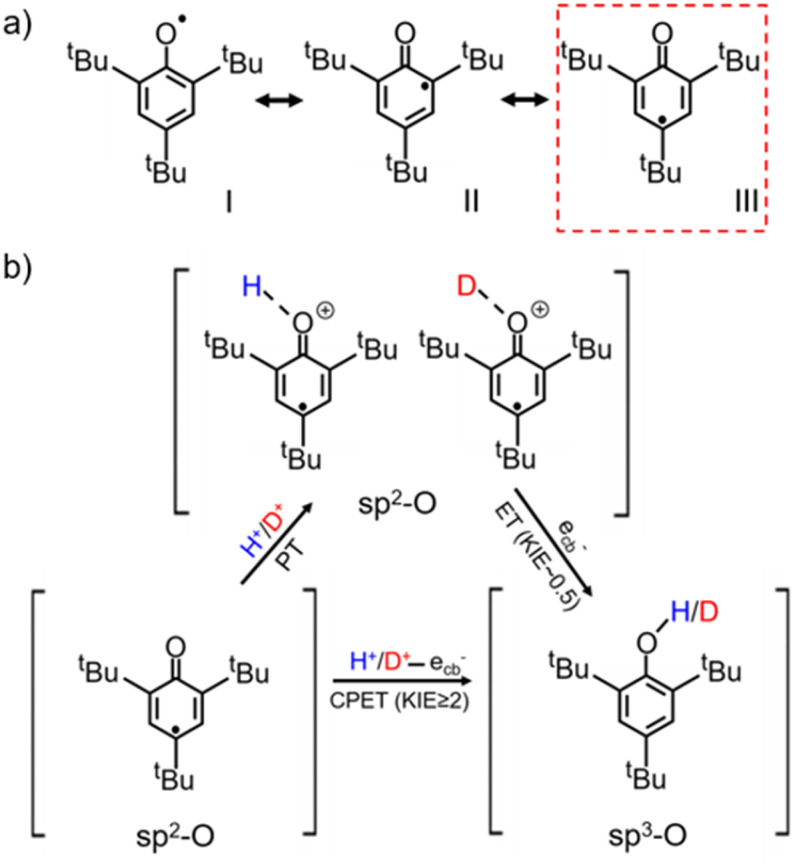
(a) Configurations of the ^*t*^Bu_3_ArO· resonance structure with type III as the major contributor;^27^ (b) schematic diagram of the CPET and PT/ET reaction pathways of the single-proton/single-electron transfer on TiO_2_ with ^*t*^Bu_3_ArO· as the acceptor.

The water-determined KSIE (H/D) reversal directly reflects the change of the photo-induced PCET reaction pathway on TiO_2_ nanoparticles. Typically, the ^*t*^Bu_3_ArO· radical has three possible configurations, as shown in Scheme 2a. Type I is the O–C single bond radical structure, while type II and type III are O=C double bond structures. Based on the reported single-crystalline structure analysis, type III is the major contributor to the stable ^*t*^Bu_3_ArO· radical, *i.e.*, in the O=C double bond configuration.^27^ Therefore, the single-proton/single-electron transfer of ^*t*^Bu_3_ArO· → ^*t*^Bu_3_ArOH is accompanied by the dissociation of the C=O double bond and the hybrid configuration transition of O=C sp^2^ → O–C sp^3^. The inverse KIE from sp^2^–sp^3^ hybridization is a very classic phenomenon in reaction kinetics.^25,28^ The secondary KIE is mainly due to the difference in bending vibrations. Due to symmetry, the in-plane and out-of-plane bending of sp^3^ hybridized carbon atoms are equivalent. The in-plane bending vibrations of sp^2^ hybridized carbon or oxygen atoms are stiffer than out-of-plane bending vibrations (there is a minor site resistance for out-of-plane bending), leading to a more significant difference in zero-point energy in the O–H and O–D bonding in the reaction of the hybrid state change. In the sp^3^ state as the substrate, the O–D bond has lower zero-point energy and needs to cross a larger energy barrier to reach the intermediate state, while in the sp^2^ state it is the opposite (Fig. S18†). In this process, an inverse KIE (H/D) can only occur when the protonated intermediate of H^+^/D^+^–O=C is formed and subsequently converted to the H/D–O–C product by the further e_cb_^−^ transfer (Scheme 2b), reflecting a stepwise PT/ET pathway at low water content (Scheme 2b). Conversely, at high water content, ^*t*^Bu_3_ArO· was directly reduced to ^*t*^Bu_3_ArOH through one-step CPET without forming such H^+^/D^+^-O sp^2^ protonated intermediates, resulting in a normal KIE ≥ 2 from the direct bond-dissociation of protons from bridging-O sites on the TiO_2_ surface (Scheme 2b).^29–31^ However, due to the high electronegativity and coordination limits of the adjacent N atoms that hinder the transfer of the unpaired electron from the O atom to the carbon ring, TEMPO as another single-proton/single-electron acceptor always retains the N–O single bond free radical structure without the O=C → O–C transition during the reaction. Thus, even with the PT intermediate formed at low water content, a KIE ∼1 rather than an inverse KIE should be observed (Scheme S2†), entirely consistent with our experimental results (Fig. 1b). Our KSIE experiments show that the participation of water can directly switch the photo-induced PCET pathway on the TiO_2_ nanoparticle catalyst from water-free stepwise PT/ET to water-participated CPET.

### *In situ* ATR-FTIR experiments

Isotopic-labeled ATR-FTIR spectroscopy was further used to *in situ* observe water molecules on the TiO_2_ surface and their interaction with photo-induced protons/electrons under continuous UV irradiation. Setting the equilibrium conditions as the blank background, a positive or negative IR response reflects the intermediate species' gradual gain or loss at the methanol-*d*_0_/*d*_4_ (CH_3_OH/CD_3_OD)/H_2_O/TiO_2_ interface during the reaction. Before measurements, TiO_2_ nanoparticles, as the typical catalyst sample, were first calcined at 773 K for 30 minutes and then cooled to room temperature under a constant dried nitrogen flow. In the water-participated system, the calcined TiO_2_ sample was further treated in water vapor under a continuous nitrogen flow for 15 min, resulting in a water content of 3.80 wt%. The experiments were carried out in a nitrogen atmosphere to prevent trapped e_cb_^−^ from being quenched by oxygen.

**Fig. 2:**
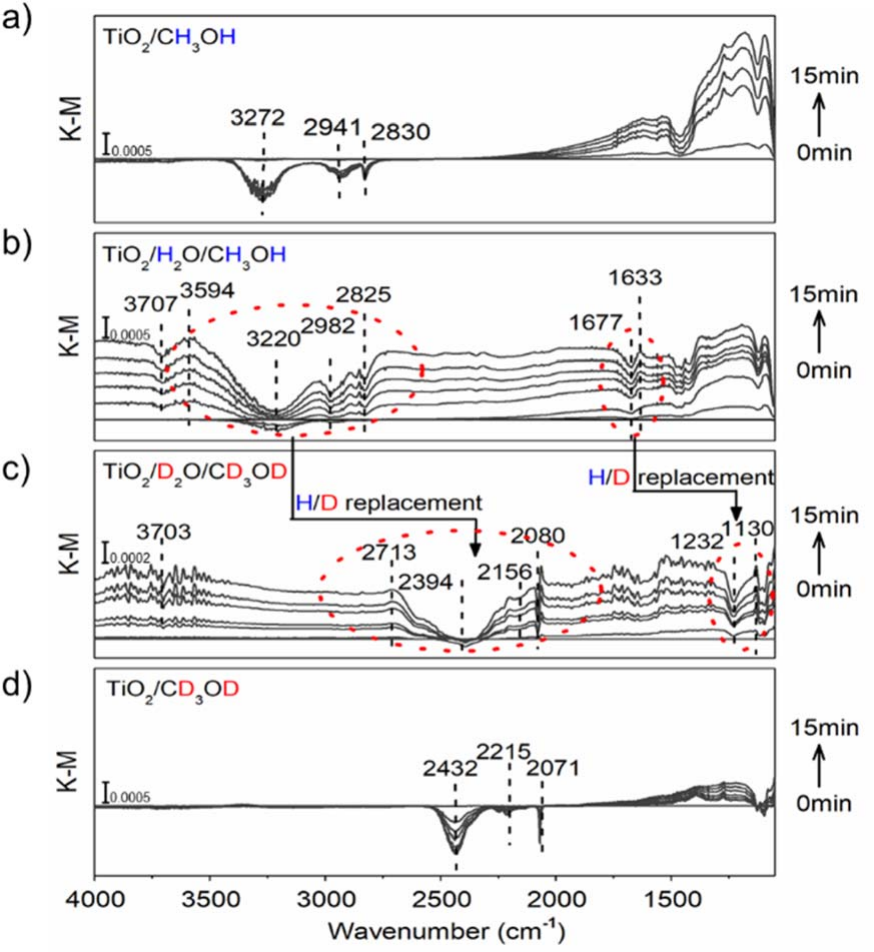
ATR-FTIR spectra *in situ* monitored at (a) TiO_2_/CH_3_OH; (b) TiO_2_/H_2_O/CH_3_OH; (c) TiO_2_/D_2_O/CD_3_OD; and (d) TiO_2_/CD_3_OD interface under constant 365 nm (3 W, LED) irradiation.

As shown in Fig. 2a, when TiO_2_/CH_3_OH was used as the ATR-FTIR sample and irradiated *in situ* with a 365 nm LED lamp, a broad negative peak at 3272 cm^−1^ and signal bands at 2941 cm^−1^ and 2830 cm^−1^ emerged from the background and increased in intensity with increasing irradiation time, corresponding to the stretching vibration signals of O–H and C–H in methanol and representing the oxidation of methanol. This is further verified by the ground-state signal on the TiO_2_/CH_3_OH system. (Figure S20†). Moreover, a strong baseline upshift absorption band from 2500 cm^−1^ to 1100 cm^−1^ was observed, which is consistent with our previous observations and can be ascribed to the absorption of trapped e_cb_^−^ on the TiO_2_ nanoparticles.^17,26,32,33^ These trapped e_cb_^−^ were localized in the surface defect layer in the form of four-coordinated Ti_4c_^3+^ under the stabilization of protons provided by the methanol oxidation.^8,17,31^ When the water vapor was first introduced to TiO_2_ nanoparticles, a broad negative absorption of O–H at 3220 cm^−1^ and a mild negative peak at 1677 cm^−1^ corresponding to the water molecule H–O–H bending vibration emerged at the TiO_2_/H_2_O/CH_3_OH interface under continuous UV irradiation. These signals are accompanied by the methanol oxidation and the baseline upshift from the absorption of trapped e_cb_^−^ (Fig. 2b), further verified by the ground-state water and methanol signal on TiO_2_ (Figure S20†). More importantly, a positive peak at 1633 cm^−1^ corresponding to the H–O–H bending vibration with a lower frequency (from 1677 cm^−1^) and a positive absorption band of O–H stretching vibration at 3594 cm^−1^ with a higher frequency (from 3220 cm^−1^) emerged. These vibrations reflect a strong interaction between water molecules and protons/e_cb_^−^ from methanol oxidation on TiO_2_ nanoparticles. Such a strong interaction causes the stretching vibration of the water to be faster while the bending vibration slows down due to the steric effect. All these effects manifested as a change in infrared absorption wavenumbers. The negative peak at 3707 cm^−1^ corresponds to the terminal hydrogen on TiO_2_.^32–34^ Moreover, when the deuterium-labeled TiO_2_/D_2_O/CD_3_OD sample is used, the strong interaction between water and protons remains. The O–H stretching vibration peak shifts towards higher frequencies (2394 cm^−1^ → 2713 cm^−1^) and H–O–H bending vibration towards lower frequencies (1232 cm^−1^ → 1130 cm^−1^) in terms of H/D replacement effects (Fig. 2c), which is consistent with the ground-state methanol-d_4_ and D_2_O signals on TiO_2_ (Figure S20†). The displacement of the deuterium-labeled proton peak indicates that the proton peak is indeed produced by the interaction of water and protons. In water-free systems, similar peak shift phenomena were no longer observed on TiO_2_/CH_3_OH or TiO_2_/CD_3_OD samples (Fig. 2a and d).

To further confirm that the observed peak shift was due to the interaction between water molecules and proton/e_cb_^−^ on the TiO_2_ surface, hydrochloric acid was used as an external proton donor to replace methanol, forming an H_2_O/HCl/TiO_2_ interface (pH = 6). When the light was off, the addition of HCl only induced mild water desorption from the TiO_2_ surface with negative peaks at 3377 cm^−1^ and 1644 cm^−1^, and no positive peaks were observed (Figure S19b†), indicating that water molecules were hardly affected by only the presence of additional protons. However, when the light was on, the negative peak at 1704 cm^−1^ and positive peak at 1663 cm^−1^ that correspond to the shift of H–O–H bending vibration towards lower frequencies were immediately observed, as well as the negative band at 3333 cm^−1^ and the emerging positive band at 3555 cm^−1^ that correspond to the shift of O–H stretching vibration towards higher frequencies (Figure S19d†), which is identical to the observed phenomenon at the TiO_2_/H_2_O/CH_3_OH interface. Moreover, a broad negative band at around 2964 cm^−1^ emerged, which can be ascribed to the destruction of hydrogen-bonds between water molecules, further demonstrating the changed water molecules in the presence of protons and photo-induced e_cb_^−^. Without the addition of HCl, no other peaks appeared except for the molecular water H–O–H bending vibration at 1640 cm^−1^ and the molecular water O–H stretching vibration at 3210 cm^−1^ under constant irradiation (Figure S19a†). We further reduced pH to 5 and repeated the ATR-FTIR experiment. As shown in Figure S19e,† the observed phenomenon is consistent with pH = 6. Furthermore, it offers a more significant blueshift signal of –OH stretching vibration and a redshift signal of H–O–H bending vibration. Such a result indicates that high proton concentrations accelerate the formation of hydrated protons, which is consistent with our conclusion. Our ATR-FTIR results solidly confirm a strong interaction between water and protons/e_cb_^−^ intermediates on the TiO_2_ surface, possibly achieved by destroying hydrogen bonds between water or forming hydrated protons^35^ which would undoubtedly affect the subsequent PCET dynamics.

Typically, protons tend to be localized at bridging –O atoms on TiO_2_ nanoparticles. At the same time, the trapping of e_cb_^−^ at neighboring Ti atoms on TiO_2_ nanoparticles under UV in the presence of methanol to quench photo-induced holes occurs.^31^ In that case, a stepwise PT/ET route with the formation of high-energy protonation intermediates was inevitable. However, according to our observations in this work, surface protons/e_cb_^−^ can form a strong interaction with water (in the form of hydrogen bonds or hydrated protons), serving as a reaction relay to favor the concerted transfer of proton/e_cb_^−^ pairs by avoiding the formation of high-energy intermediates. In other words, the presence of water lowered the reaction energy barrier of photo-induced PCET on TiO_2_ nanoparticles by switching the reaction pathway from stepwise PT/ET to CPET.

### DFT calculations

**Fig. 3:**
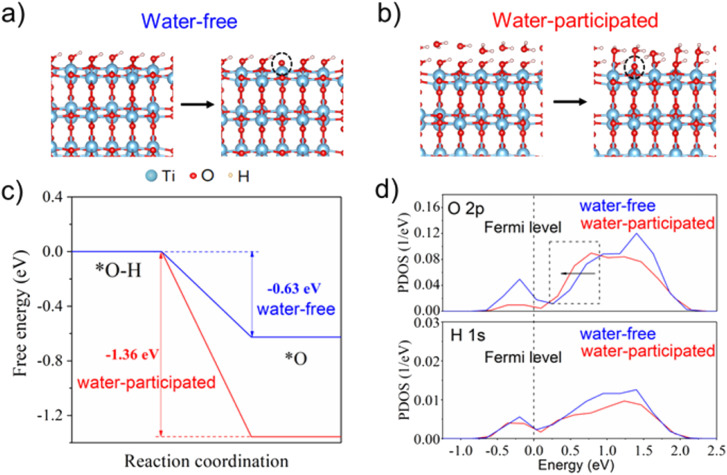
Redox reactions were simulated on (a) water-free system and (b) water-participated system. (c) Free energy profiles of hydrogen in both water-participated/water-free systems at pH = 7 and *U* = 0 V *vs.* SHE; and (d) PDOS of the surface-OH group in both water-participated/water-free systems. The dashed line stands for the Fermi level.

DFT calculations were further employed to investigate the impact of water molecules on the single-proton/single-electron transfer on the TiO_2_ surface (for details of the computational methods, see the ESI†). As shown in Fig. 3a, the calculated free energy profiles of electron and proton transfer suggest that the existence of water molecules on the TiO_2_ surface can significantly promote the transfer of electrons and protons from the surface –OH group due to more negative reaction energy (−1.36 eV) in comparison with the water-free system (−0.63 eV). The projected density of states (PDOS) profiles of the surface –OH group further indicate that the edge line of the main peak of O 2p states in the –OH group with a water layer on the TiO_2_ surface is closer to the Fermi level than that without a water layer, which is often active in the catalysis reaction. This also suggests the higher reaction activity of the –OH group in the presence of a water layer on the TiO_2_ surface (Fig. 3b). As a result, the surface –OH group with neighboring water is more active than that without water, which coincides with our experimental observations.

## Conclusion

An unexpected water-controlled switch of the photo-induced single-proton/single-electron transfer pathway on TiO_2_ nanoparticles was discovered. On dried TiO_2_ nanoparticles (water content <1.7 wt%, ∼monolayer coverage), the photo-induced PCET follows a stepwise PT/ET pathway with the formation of a high-energy protonation intermediate, resulting in an inverse KSIE (H/D) ∼0.5 with ^*t*^Bu_3_ArO· and KSIE (H/D) ∼1 with TEMPO in methanol-*d*_0_/*d*_4_ systems. However, in the presence of a trace amount of water (>2 wt% in TiO_2_, ∼two layer coverage), the single-proton/single-electron transfer reaction on TiO_2_ nanoparticles follows a concerted pathway with a lower energy barrier, leading to a normal KSIE (H/D) ≥2 with both reagents. Our work is the first to clarify the genuine impact of water on the PCET pathway responsible for the performance enhancement in water-participated systems in terms of PCET, which dispels previous confusion on this issue and can be used for the design of efficient semiconductor-based photocatalytic systems.

## Data availability

The data supporting the findings of this study are available within the article and its ESI† files. All other relevant source data are available from the corresponding author upon request following the data management specifications of Jiangsu University and University of Michigan. Source data are provided with this paper.

## Author contributions

Z. Liu: investigation, data curation, and writing of the original draft. W. Peng, Y. Lin, X. Lin, S. Yin, S. Jia, and D. Ma: resources. Y. Yan and P. Zhou: writing-review. Y. Yan, W. Ma, and J. Zhao: conceptualization, supervision, writing-review and editing.

## Conflicts of interest

There are no conflicts to declare.

## Supplementary Material

SC-014-D2SC07038C-s001
