# Supplementary material for: Water molecule switching heterogeneous proton-coupled electron transfer pathway
Source: Chem Sci. 2023 Apr 3;14(17):4564–70. doi: 10.1039/d2sc07038c (PMC10155922; doi:10.1039/d2sc07038c)
Supplement: SC-014-D2SC07038C-s001 [file SC-014-D2SC07038C-s001.pdf]

Electronic Supplementary Material (ESI) for Chemical Science.
This journal is © The Royal Society of Chemistry 2023

Supporting information

Water Molecule Switching Heterogeneous Proton-Coupled Electron Transfer Pathway

Zhonghuan Liu[b], Wei Peng[a], Yuhan Lin[a], Xinyu Lin[b], Shikang Yin[b], Shuhan Jia[b], Dongge Ma[d], Yan Yan[b,*], Peng Zhou[c,*], Wanhong Ma[a,*], Jincai Zhao[a]

[a] *Key Laboratory of Photochemistry, Beijing National Laboratory for Molecular Sciences, Institute of Chemistry, Chinese Academy of Sciences, Beijing, 100190, PR China*

[b] *Institute of Green Chemistry and Chemical Technology, School of Chemistry and Chemical Engineering, Jiangsu University, Zhenjiang 212013, PR China*

[c] *Department of Electrical Engineering and Computer Science, University of Michigan, Ann Arbor, MI 48109, USA*

[d] *Department of Chemistry, College of Chemistry and Materials Engineering, Beijing Technology and Business University, Beijing 100048, China*

Corresponding authors:

W. Ma (whma@iccas.ac.cn)

Y. Yan (dgy5212004@163.com)

P. Zhou (dpzhou@umich.edu)

Supplementary Methods

Materials

Commercial titanium dioxide nanoparticles (TiO_2, 30 nm, anatase), deuterium oxide (D_2O, 99 atom % D), and methanol-d_4 (CD_3OD, 99.8% D) were purchased from Sigma-Aldrich Company Ltd. Methanol (CH_3OH) was purchased from Sinopharm Chemical Reagent Co. Ltd. The toluene-d_8 used in ^1H NMR experiments was from Innochem. 2,4,6-tri-tert-butylphenol (97%) was purchased from Acros. The corresponding phenoxyl radical $^tBu_3ArO\cdot$ was prepared according to the reference.[1] All reagents used in the synthesis were analytical grade without further purification. Deionized water, with a resistivity of 18 MΩcm, was used throughout the experiments.

Preparation of TiO_2 nanoparticles with different water content

In a typical preparation procedure, untreated TiO_2 nanoparticles were calcined in a tube furnace at 773K for 30 min. After the temperature was cooled to room temperature, dried TiO_2 samples were transferred to a humidity chamber with different air humidity degrees (0%~100%) and kept for 30 minutes.

KSIE(H/D) experiments

In a typical procedure, 20 mg anatase was dispersed in 8 mL methanol-d_0. The suspension was purged by Argon for 10 min and transferred to the Ar glovebox. After that, 0.5 mL $^tBu_3ArO\cdot$ in acetonitrile of 2.5 mmol/L was added to it. Finally, the resulting suspension was sealed into a unique tube for an online ESR experiment in 90s under UV irradiation (365 nm, 68 mW/cm^2). Deuterated methanol-d_4 was used to repeat the experiment above under otherwise identical conditions. The settings for the ESR spectrometer were as follows: center field, 3400 G; sweep width, 400/800 G; microwave frequency, 9.52 GHz; field modulation frequency,100 kHz.

Fe(III)-1, 10-Phenanthroline spectrometric titration measurements.

The electron concentration of trapped electrons on reduced TiO_2 samples was measured by Fe (III)- 1, 10-phenanthroline titration spectrometric method. 1, 10-phenanthroline spectrometric measurement is a simple and widely used method for the measurement of Fe (II) ions. Here, we use the Fe (III) solution to titrate the trapped electrons on TiO_2 samples that quantitatively lead to the produce of Fe (II) ion, then used 1, 10-phenanthroline to measure the concentration of Fe (II) ions. Thus, we can quantitatively obtain the concentration of trapped electrons. The concentration of $Fe(NO_3)_3$ solutions employed in this measurement is 10^{-3} M. 0.2% 1, 10-phenanthroline water solution and pH = 4.6 HAc-NaAc buffer solution were previously prepared for use. The pH = 4.6 HAc-NaAc buffer solution was prepared by dissolving 135 g sodium acetate and 120 mL acetic acid into 500 mL water solution. Before the titration, the $Fe(NO_3)_3$ solution is purged by Argon for 30 min to remove oxygen. The titration was conducted in the Argon glove box.

In a typical procedure, 2.5 mL reduced TiO_2 solution was taken in the Argon glove box and mixed with 2.5 mL $Fe(NO_3)_3$ solution. 5 min later, the resulting mixed solution was taken out and then centrifuged to conduct the spectrometric measurement. The spectrometric measurement was conducted in air atmosphere. 1.5 mL centrifuged supernatant taken from the Argon glove box was mixed with 1.5 mL pH = 4.6 HAc-NaAc buffer solution, and then added with 1 mL 0.2% 1, 10-phenanthroline water solution to obtain a red solution. Waiting 5 minutes to obtain a stable state, the resulting solution was transferred in a quartz cuvette and measured on a Hitachi U3900 spectroscopy. The spectrum at 0 minutes was used as the background. Obtained absorbance value was compared with the standard fitting line to obtain a certain Fe (II) concentration.

DRIFTS Measurement

In-situ diffuse reflection infrared Fourier transform spectroscopy (DRIFTS) experiments were implemented on a Thermo Scientific Nicolet iS10 spectrometer equipped with a mercury cadmium telluride (MCT) detector. In a typical procedure, the catalysts were housed in Harrick Praying Mantis high-temperature reaction tank with a ZnSe window. The reaction chamber was heated to 773K, and the stabilized system was used as the background spectrum. A series module was used to observe the changes in the cooling process on the TiO_2 surface.

TGA experiments

Thermogravimetric analysis (TGA) was performed on Per-Kin-Elmer Pyris 1 and TGA 7 thermogravimetric analyzers in N_2 atmospheres. Different TiO_2 solid samples were heated from 25 °C to 700 °C at a 20 °C/min rate.

NMR ^{1}H Measurement

After reducing ^{t}Bu$_3$ArO·/TEMPO with TiO_2 methanol suspension under UV irradiation, the solvent was removed by purging with Ar. Then the residue was transferred to the Ar glovebox and dissolved in toluene-d_8 for ^{1}H-NMR measurement.

ATR-FTIR experiments

ATR-FTIR measurement was employed to detect the surface species and their changes with the in-situ reaction process on TiO_2 films. The ATR-FTIR spectroscopy experimental setup was similar to that described in the references.[2] The instrumental setup consisted of a Harrick Horizon multiple internal reflection accessories coupled to a 1 mL flow-through cell containing an AMTIR(As/Se/Ge) crystal on the bottom plate and a quartz window on the top plate. Eleven infrared bounces were allowed using a 45° internal reflection element (50×10×2 mm^3). The FT-IR measurements were performed on a Nicolet iS10 FTIR with an MCT detector. In a typical photocatalysis procedure, a layer of methanol-d_0/d_4 was dripped onto the surface of the AMTIR crystal that was coated with a TiO_2 (Wetted by water or deuterium water vapor before calcination) film. Argon degassed the apparatus for 30 min, and the crystal was then scanned to obtain the background spectrum. Time-

resolved in-situ FTIR data was then collected, turning up the 365 nm LED lamp.

Suspension degree measurement

The titanium dioxide of 20 mg is evenly scattered in methanol, and the ultrasonic is 10min evenly. The obtained solution is immediately placed in the rack. At this time, the state is set to the background, using a fiber optic photometer to measure its luminous flux, the obtained spectrum is the increase in luminous flux caused by in-situ deposition within a same measurable time.

Computational methods

The photocatalytic properties of TiO_2 were investigated by the Vienna Ab-initio Simulation Package (VASP) with the revised Perdew-Burke-Ernzerh functional of (RPBE) of the generalized gradient approximation (GGA). The Van Der Waals correction using DFT-D2 method of Grimme was considered in the calculations. The Hubbard U term (DFT+U) was added on O 2p orbitals at the value of 6.3 eV and Ti 3d orbitals at the value of 4.2 eV. The interaction between ionic core and valence electrons was obtained from PAW pseudo-potential. TiO_2 surface was described by its typical (101) facet, stimulated by the 1×3 supercell consisting of 6 O–Ti–O layers. The surface –OH group was built by bonding the surface bridge O atom with one hydrogen atom. The energy cutoff of the plane-wave basis of 400 eV and the energy convergence threshold of 1.0×10^{-5} eV were adopted in the geometry optimization at the gamma point. After geometry optimization, the projected density of states (PDOS) of the surface –OH group was calculated with the Monkhorst-Pack k-point mesh of $2 \times 2 \times 1$, energy convergence threshold of 1.0×10^{-5} eV, and the energy cutoff of the plane-wave basis of 400 eV. The calculations of Gibbs's free energy changes (ΔG) of all reaction steps adopted the reported standard hydrogen electrode (SHE) model.

Supplementary Figures

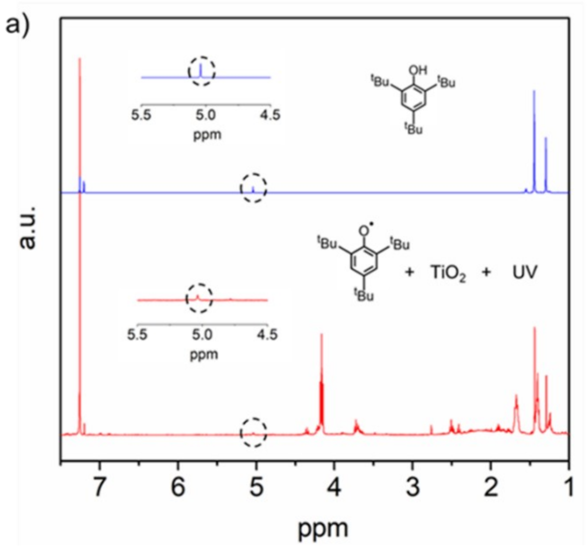

Figure S1. ^{1}H-NMR spectra of single-proton/single-electron transfer products (a) ${}^{t}Bu_3ArOH$ and (b) TEMPOH (After the reaction, the TiO_2 was filtered out, and the solvent was removed under anaerobic condition, then the residue was dissolved in toluene-d_8).

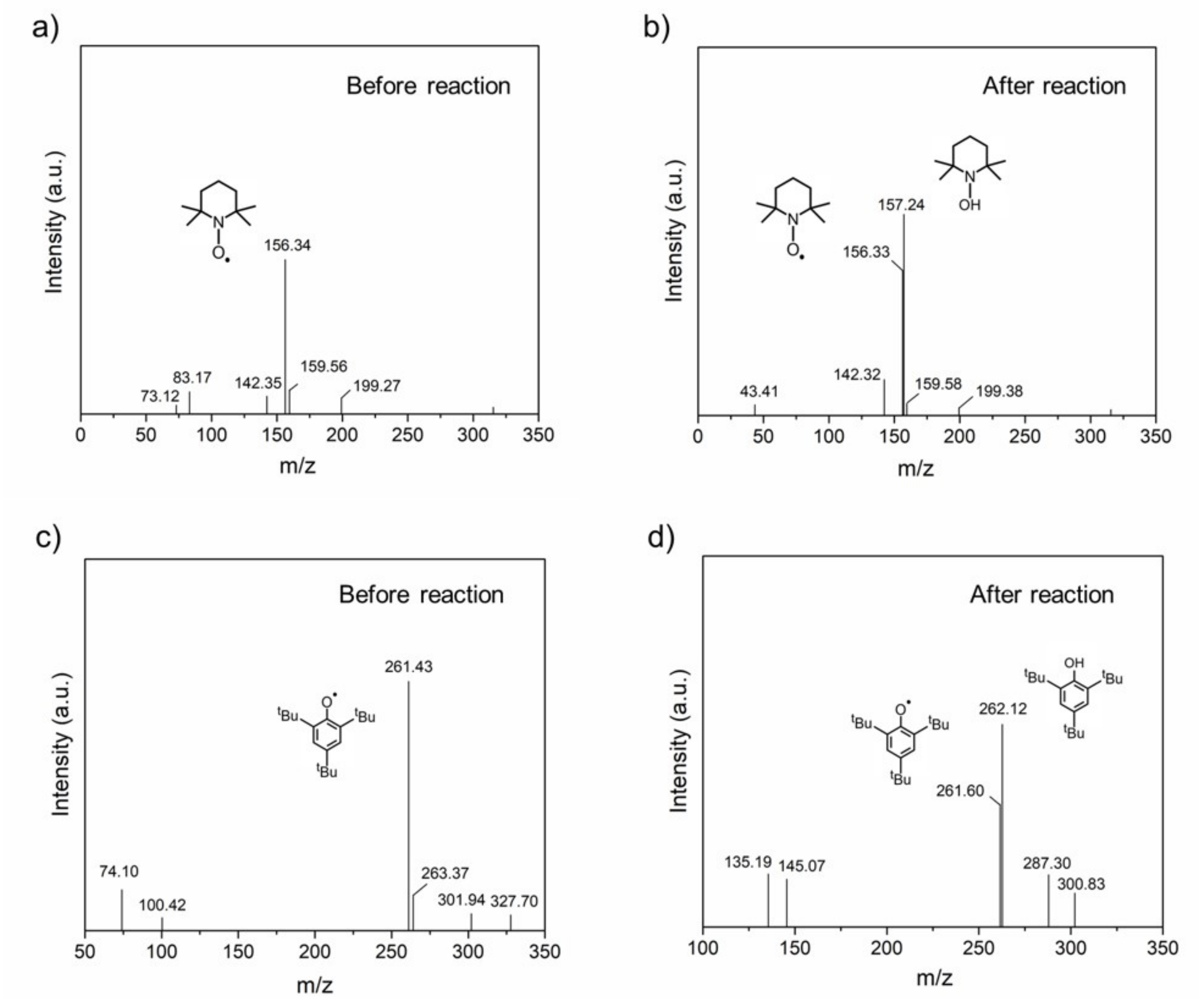

Figure S2. Comparison of Mass spectra before and after the reaction: (a) before, and (b) after reaction for TEMPO and (c), (d) were the ${}^{t}Bu_3ArO\cdot$.

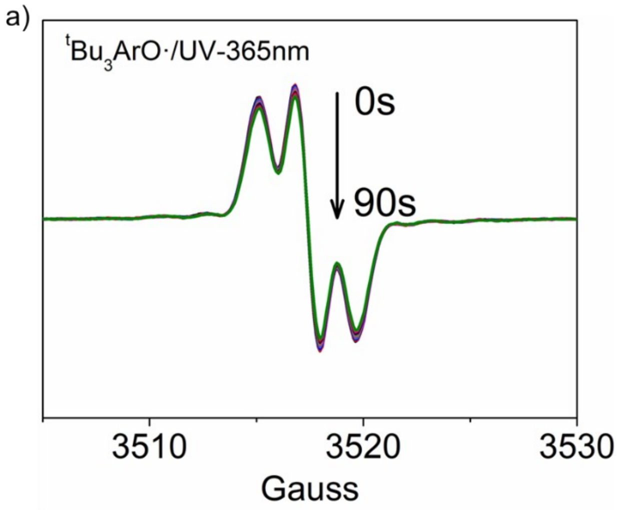

Figure S3. Control experiments without TiO_2 catalysts with (a) $^tBu_3ArO\cdot$ or (b) TEMPO as the substrate.

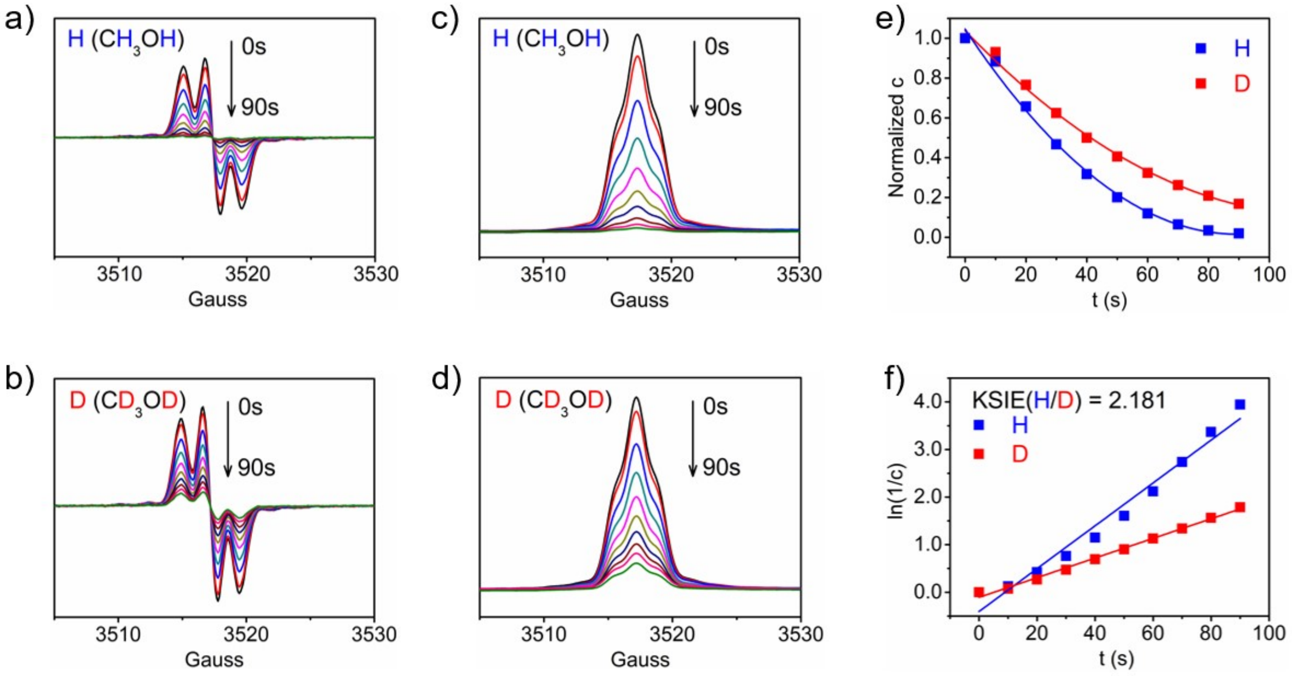

Figure S4. ESR spectra in-situ monitor the concentration profiles of $^tBu_3ArO\cdot$ under white light irradiation (300 W, Xe lamb) with pristine TiO_2 as a catalyst in (a) methanol-d_0 and (b) methanol-d_4. KSIE (H/D) was determined by the (c) (d) integration of EPR profiles within 90 s in 10 s-interval. KSIE value was given by comparing the (e) (f) reaction kinetics in H/D systems.

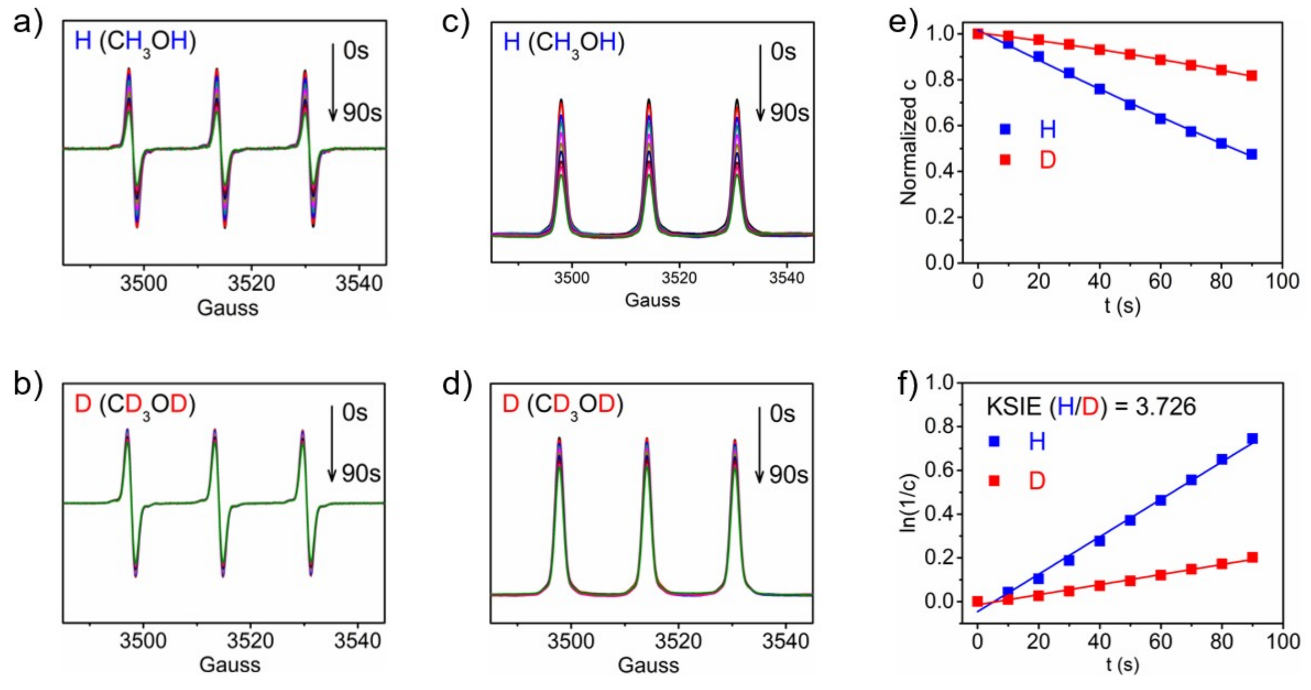

Figure S5. ESR spectra in-situ monitor the concentration profiles of TEMPO under the 90 s of white light irradiation (300 W, Xe lamb) with pristine TiO_2 as a catalyst in (a) methanol-d_0 and (b) methanol-d_4. KSIE (H/D) was determined by the (c) (d) integration of EPR profiles within 90 s in 10 s-interval. KSIE value was given by comparing the (e) (f) reaction kinetics in H/D systems.

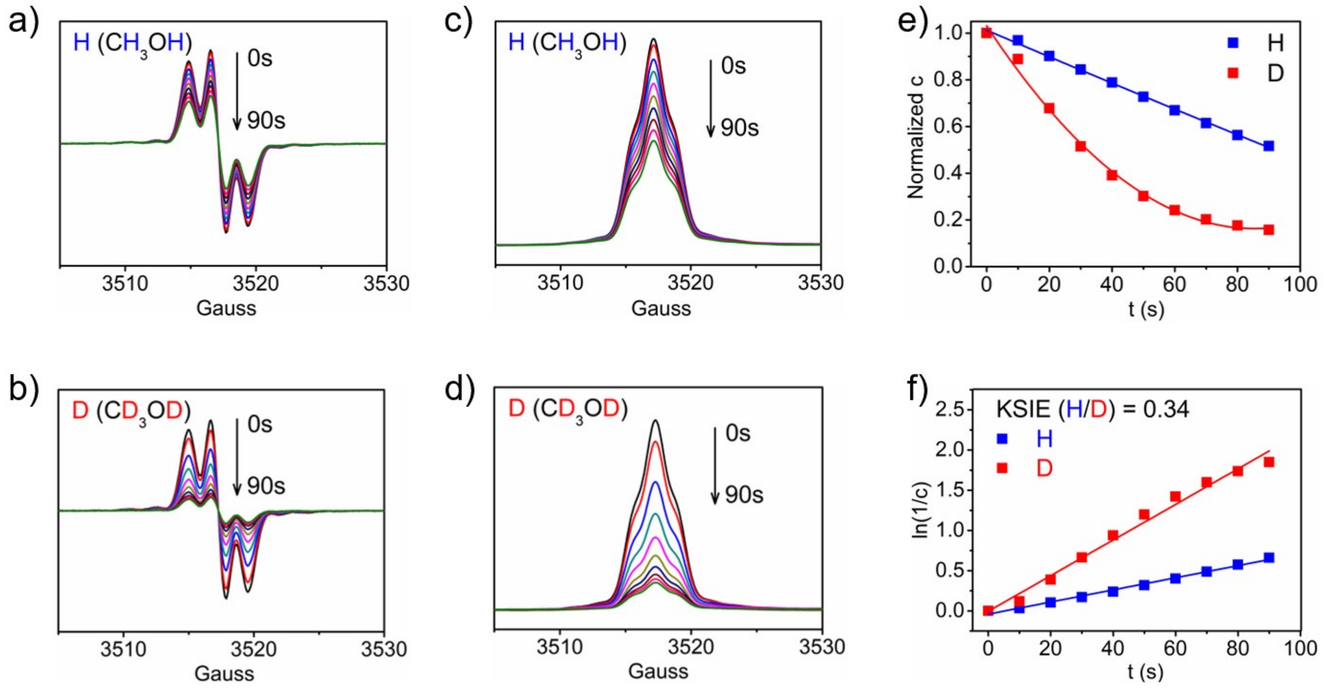

Figure S6. ESR spectra in-situ monitor the concentration profiles of $^{t}Bu_3ArO\cdot$ under the 90 s of white light irradiation (300 W, Xe lamb) with dried TiO_2 after 773 K calcination as a catalyst in (a) methanol-d_0 and (b) methanol-d_4. KSIE (H/D) was determined by the (c) (d) integration of EPR profiles within 90 s in 10 s-interval. KSIE value was given by comparing the (e) (f) rection kinetics in H/D systems.

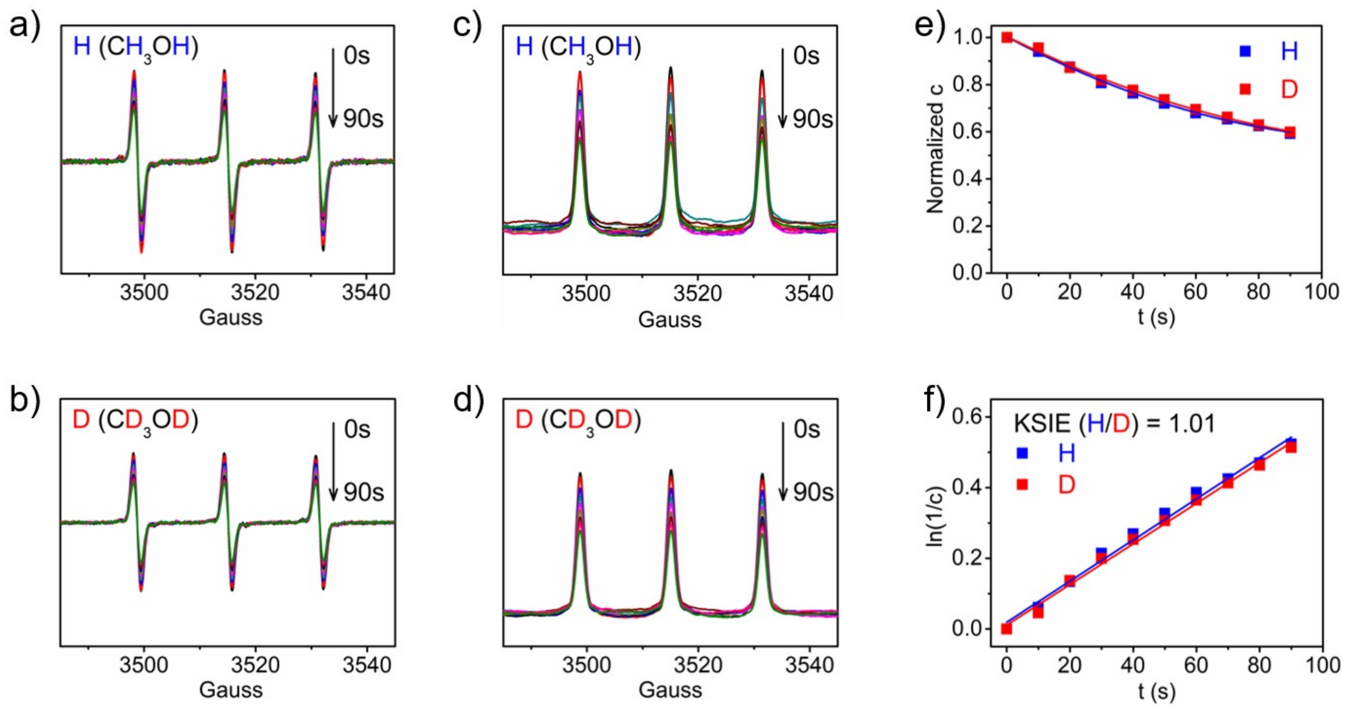

Figure S7. ESR spectra in-situ monitor the concentration profiles of TEMPO under the 90 s of white light irradiation (300 W, Xe lamb) with dried TiO_2 after 773 K calcination as a catalyst in (a) methanol-d_0 and (b) methanol-d_4. KSIE (H/D) was determined by the (c) (d) integration of EPR profiles within 90 s in 10 s-interval. KSIE value was given by comparing the (e) (f) rection kinetics in H/D systems.

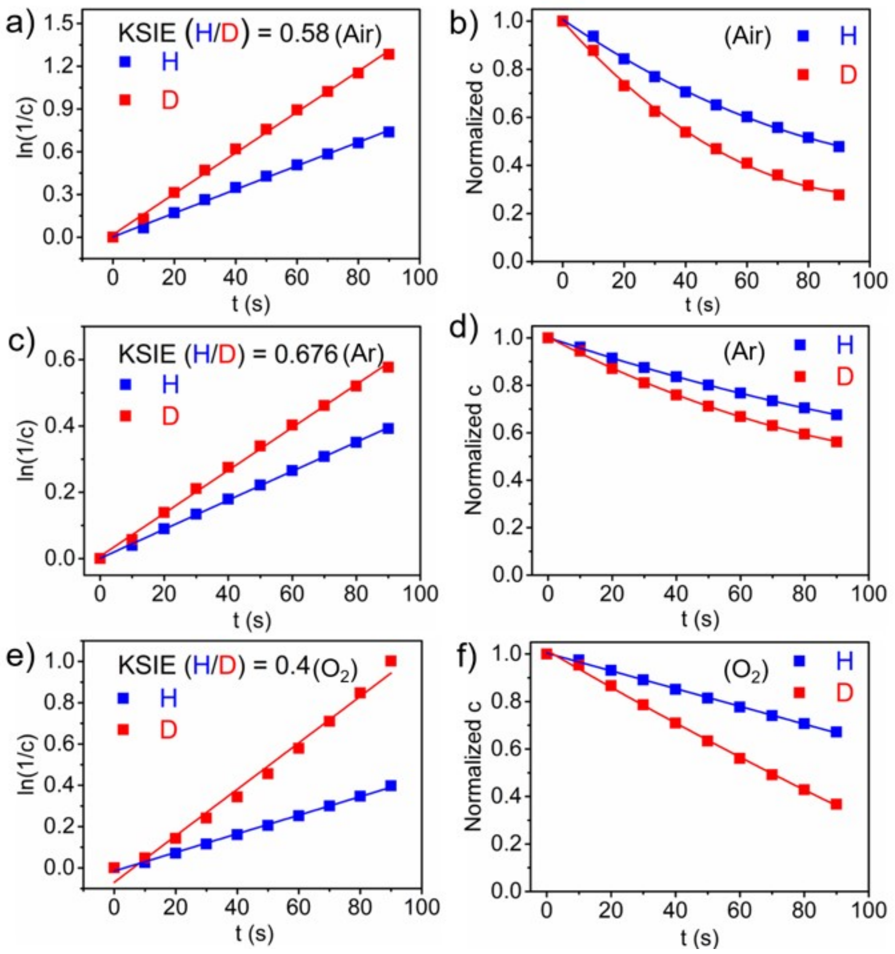

Figure S8. Kinetic profiles of dried TiO_2 nanoparticles calcined in different atmospheres with ${}^{t}Bu_3ArO\cdot$ as substrate and corresponding KSIE value. (a) (b) Air calcined; (c) (d) Ar calcined; and (e) (f) O_2 calcined.

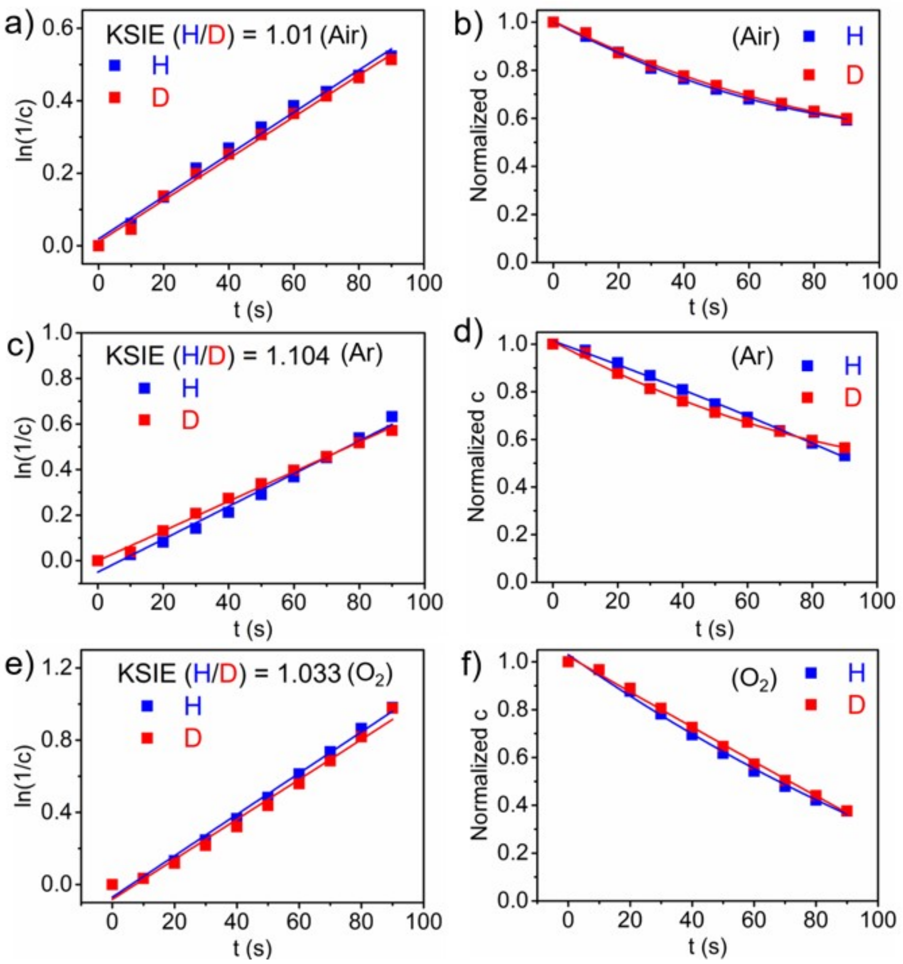

Figure S9. Kinetic profiles of dried TiO_2 nanoparticles calcined in different atmospheres with $^tBu_3ArO\cdot$ as substrate and corresponding KSIE value. (a) (b) Air calcined; (c) (d) Ar calcined; and (e) (f) O_2 calcined.

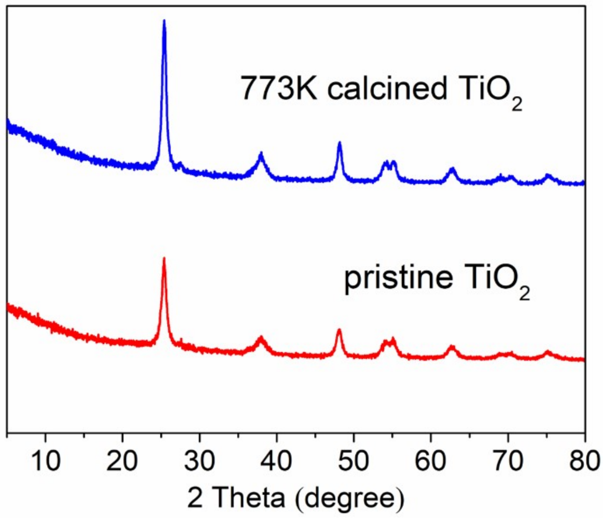

Figure S10. XRD spectra of untreated TiO_2 and 773 K calcined TiO_2 nanoparticles.

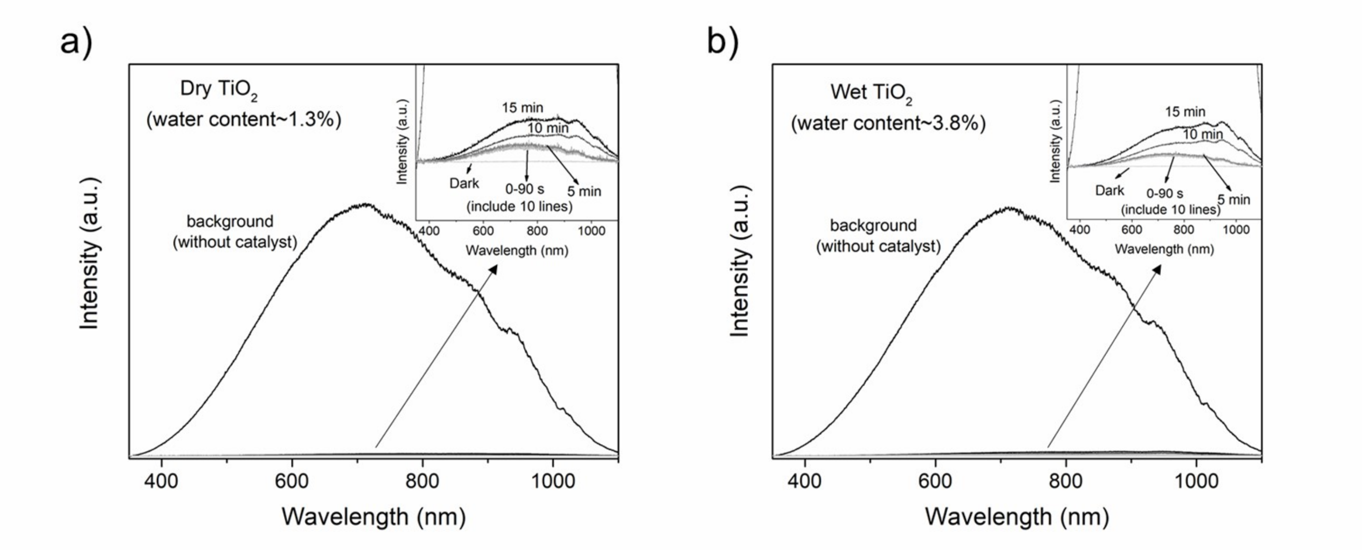

Figure S11. The luminous flux at the same settling time in the in-situ state measured by the fiber optic photometer (a) dry TiO_2 (water content~1.3%) and (b) wet TiO_2 (water content~3.8%).

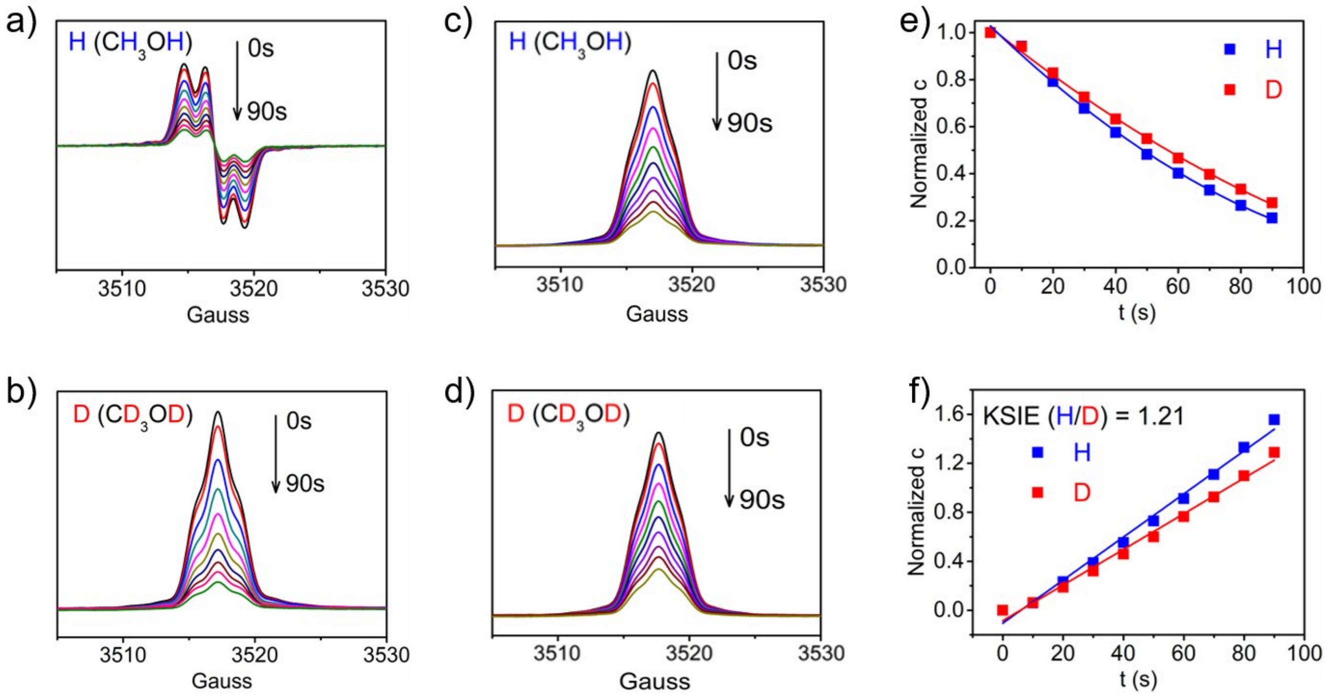

Figure S12. ESR spectra in-situ monitor the concentration profiles of ${}^{t}Bu_3ArO\cdot$ under the 90 s of white light irradiation (300 W, Xe lamb) with TiO_2 after 773K calcination and then kept in ambient condition for a week as a catalyst in (a) methanol-d_0 and (b) methanol-d_4. KSIE (H/D) was determined by the (c) (d) integration of EPR profiles within 90 s in 10 s-interval. KSIE value was given by comparing the (e) (f) rection kinetics in H/D systems.

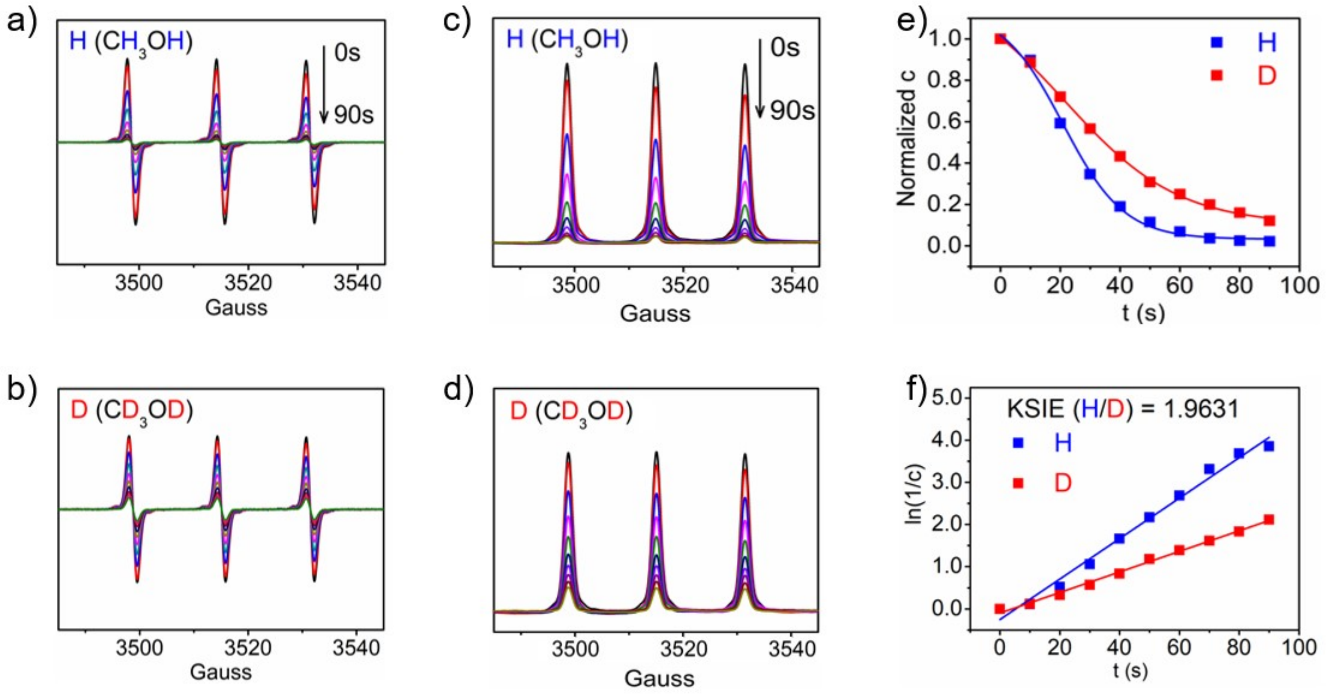

Figure S13. ESR spectra in-situ monitor the concentration profiles of TEMPO under the 90 s of white light irradiation (300 W, Xe lamb) with TiO_2 after 773 K calcination and then kept in ambient condition for a week as a catalyst in (a) methanol-d_0 and (b) methanol-d_4. KSIE (H/D) was determined by the (c) (d) integration of EPR profiles within 90 s in 10 s-interval. KSIE value was given by comparing the (e) (f) rection kinetics in H/D systems.

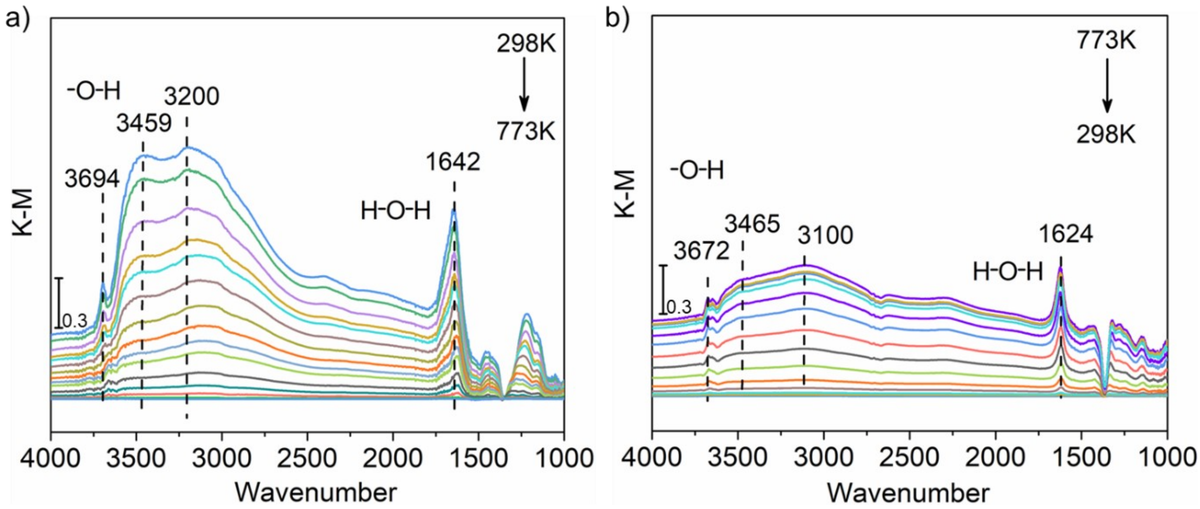

Figure S14. In-situ DRIFT spectra to observe the change of surface water adsorption on TiO_2 nanoparticles during (a) heating process from 298 K to 773 K and (b) colling process from 773 K to 298 K. The equilibrium state at 773 K was used as a blank background baseline.

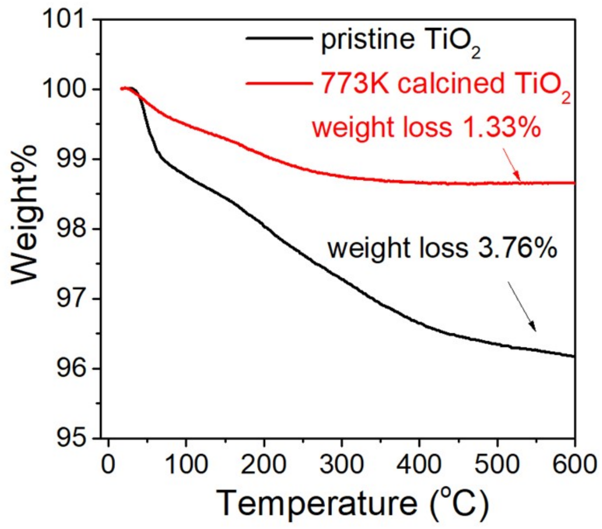

Figure S15. Thermogravimetric analysis (TGA) spectra to determine the water content in pristine TiO_2 and the 773 K calcined counterpart.

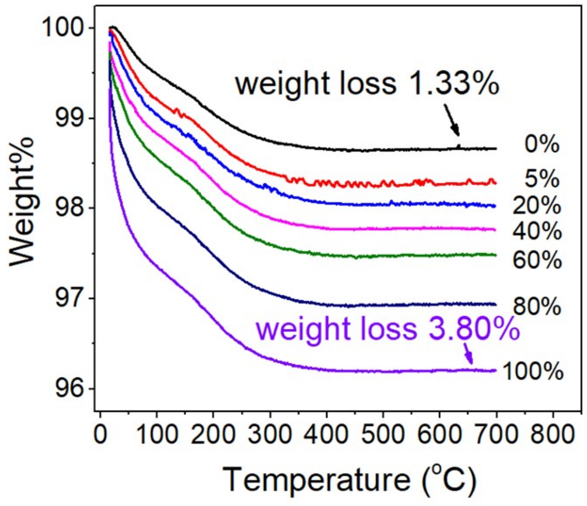

Figure S16. Thermogravimetric analysis (TGA) spectra to determine the water content in TiO_2 after being treated in humid conditions (0%-100%) for 30 min.

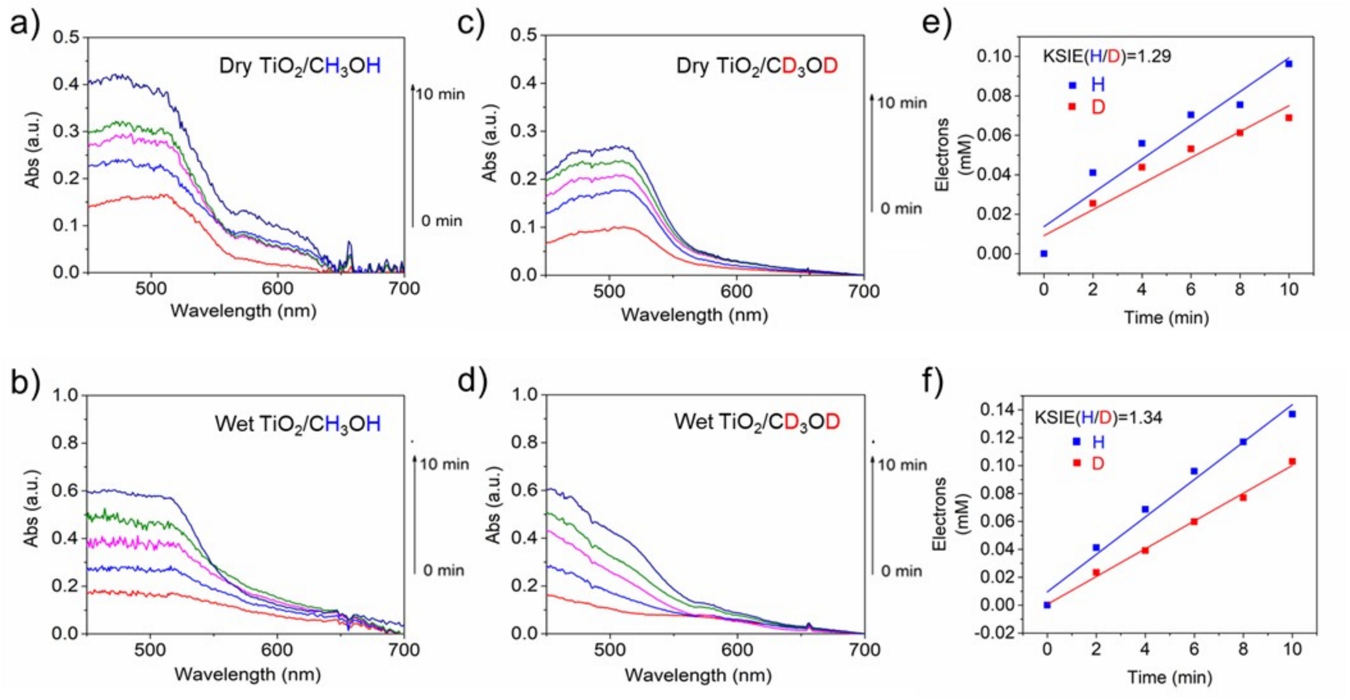

Figure S17. Control experiments of Fe(III)-1,10-Phenanthroline titration measurements. The UV/Vis absorbance spectra collected during titration of the trapped electron samples with different light-irradiation time were carried out on (a) dry TiO_2 (water content~1.3%) -CH_3OH; (c) dry TiO_2 (water content~1.3%) -CD_3OD; (b) wet TiO_2 (water content~3.8%) -CH_3OH; (d) wet TiO_2 (water content~3.8%) -CD_3OD, and the KIE value obtained by electron number fitting were (e) dry TiO_2, (f) water treatment TiO_2, background electrons before 0 min were deducted.

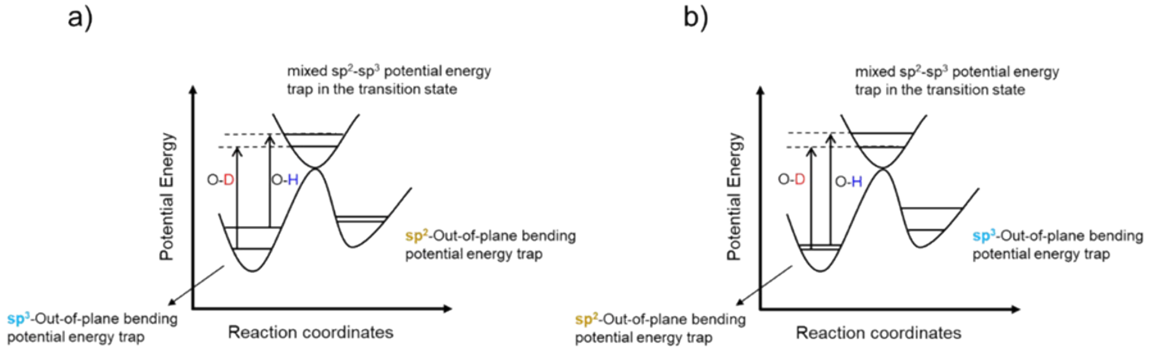

Figure S18. (a) Origin of the normal secondary KIE and (b) origin of the inverse secondary KIE.

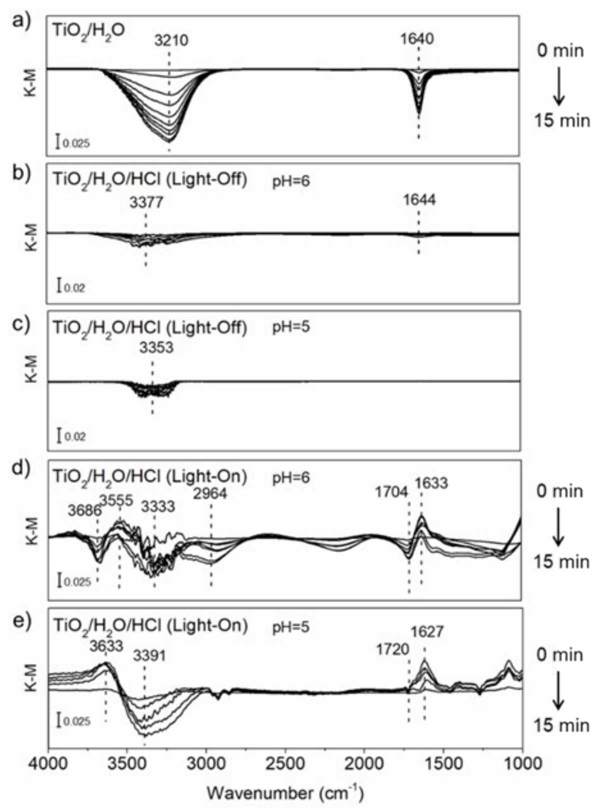

Figure S19. ATR-FTIR spectra in-situ monitored at (a) TiO_2/H_2O interface under constant 365 nm (3 W, LED) irradiation; (b) $TiO_2/H_2O/HCl$ (pH=6) without UV irradiation; (c) $TiO_2/H_2O/HCl$ (pH=5) without UV irradiation; (d) $TiO_2/H_2O/HCl$ (pH=6) interface under constant 365 nm (3 W, LED) irradiation; and (e) $TiO_2/H_2O/HCl$ (pH=5) interface under constant 365 nm (3 W, LED) irradiation.

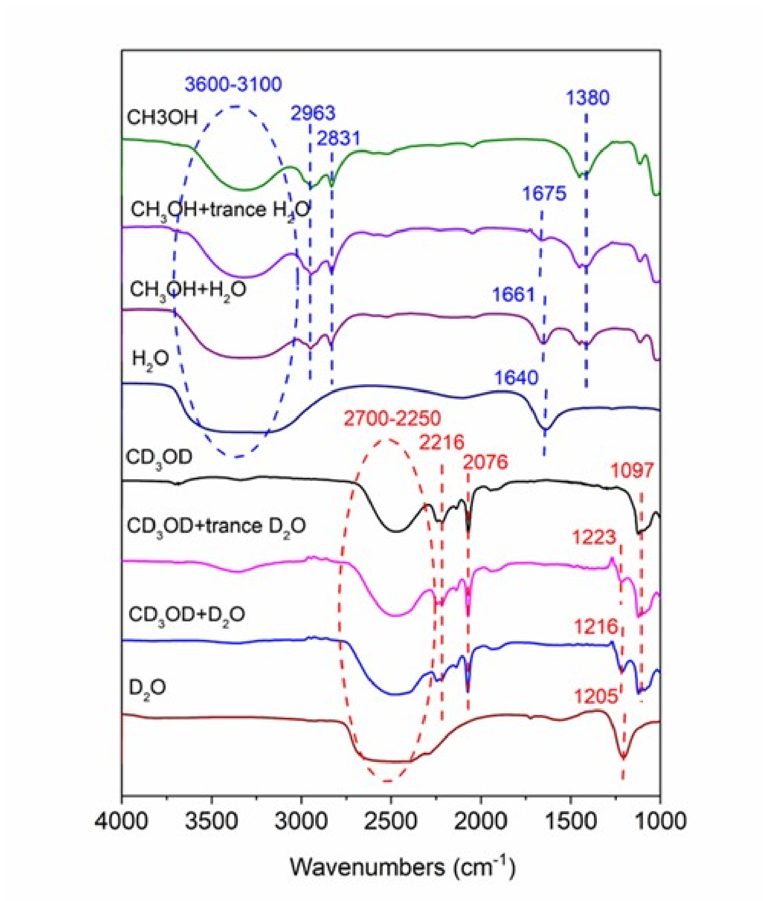

Figure S20. ATR-FTIR ground-state spectroscopy based on TiO_2 thin films.

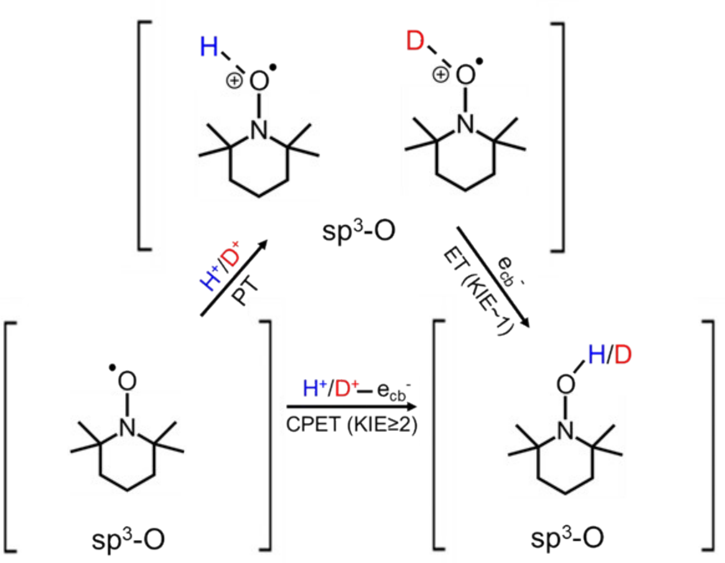

Scheme S1. Schematic diagram of CPET and PT/ET reaction pathways of the single-proton/single-electron transfer on TiO_2 with TEMPO as the acceptor.

	Crystal surface	FWHM	2 Theta	Theta	D
Pristine TiO_2	101	0.6394	25.5	12.75	13.35
	004	1.4801	37.9	18.95	5.95
	200	0.7262	48.2	24.10	12.56
773K calcined TiO_2	101	0.7344	25.5	12.75	11.63
	004	1.6089	37.9	18.95	5.47
	200	0.8481	48.2	24.10	10.76

Table S1. Crystallite diameter obtained by Scherrer analysis under pristine and 773 K calcined TiO_2.

**Water content (wt%)**	**Calculated water coverage (layers)**
1.377	1.096
1.713	1.408
1.969	1.621
2.237	1.842
2.517	2.072
3.067	2.525
3.795	3.124

Table S2. The calculated water coverage for the different TiO_2 samples (with water content of 1.34wt%~3.80wt%).

Quantum efficiency under different reaction conditions.

On 773 K calcinated TiO_2 nanoparticles:

$H(CH_3OH)$:

$$N = \frac{E\lambda}{hc} = \frac{68 \times 1 \times 10^{-3} \times 90 \times 365 \times 10^{-9}}{6.626 \times 10^{-34} \times 3 \times 10^{8}} = 1.12 \times 10^{19}$$

$$AQY = \frac{the\ number\ of\ reduced\ {}^{t}Bu_3ArO\cdot\ molecules}{1.12 \times 10^{19}} \quad (1)$$

$$= \frac{6.02 \times 10^{23} \times 6.15 \times 10^{-7}}{1.12 \times 10^{19}} = 3.31\%$$

$D(CD_3OD)$:

$$N = \frac{E\lambda}{hc} = \frac{68 \times 1 \times 10^{-3} \times 90 \times 365 \times 10^{-9}}{6.626 \times 10^{-34} \times 3 \times 10^{8}} = 1.12 \times 10^{19}$$

$$AQY = \frac{the\ number\ of\ reduced\ {}^{t}Bu_3ArO\cdot\ molecules}{1.12 \times 10^{19}} = \frac{6.02 \times 10^{23} \times 10.75 \times 10^{-7}}{1.12 \times 10^{19}} = [illegible] \quad (2)$$

KIE(H/D) from AQY is KIE(H/D) = AQY(H)/AQY(D)= 0.57

On pristine TiO_2 nanoparticles:

$H(CD_3OH)$:

$$N = \frac{E\lambda}{hc} = \frac{68 \times 1 \times 10^{-3} \times 90 \times 365 \times 10^{-9}}{6.626 \times 10^{-34} \times 3 \times 10^{8}} = 1.12 \times 10^{19}$$

$$AQY = \frac{the\ number\ of\ evolved\ {}^{t}Bu_3ArO\cdot\ molecules}{1.12 \times 10^{19}} = \frac{6.02 \times 10^{23} \times 12.38 \times 10^{-7}}{1.12 \times 10^{19}} = 6[illegible] \quad (3)$$

$D(CD_3OD)$:

$$N = \frac{E\lambda}{hc} = \frac{68 \times 1 \times 10^{-3} \times 90 \times 365 \times 10^{-9}}{6.626 \times 10^{-34} \times 3 \times 10^{8}} = 1.12 \times 10^{19}$$

$$AQY = \frac{the\ number\ of\ evolved\ {}^{t}Bu_3ArO\cdot\ molecules}{1.12 \times 10^{19}}$$

$$= \frac{6.02 \times 10^{23} \times 9.96 \times 10^{-7}}{1.12 \times 10^{19}} = 5.35\% \quad (4)$$

KIE(H/D) from AQY is KIE(H/D) = AQY(H)/AQY(D) = 1.24

For TEMPO reduction experiments, the AQYs in 90 seconds of reaction were calculated as follows:

On 773 K calcinated TiO_2 nanoparticles:

$H(CH_3OH)$:

$$N = \frac{E\lambda}{hc} = \frac{68 \times 1 \times 10^{-3} \times 90 \times 365 \times 10^{-9}}{6.626 \times 10^{-34} \times 3 \times 10^{8}} = 1.12 \times 10^{19}$$

$$AQY = \frac{the\ number\ of\ reduced\ TEMPO\ molecules}{1.12 \times 10^{19}} = \frac{6.02 \times 10^{23} \times 6.04 \times 10^{-7}}{1.12 \times 10^{19}} = 3.2[illegible] \quad (5)$$

$D(CD_3OD)$:

$$N = \frac{E\lambda}{hc} = \frac{68 \times 1 \times 10^{-3} \times 90 \times 365 \times 10^{-9}}{6.626 \times 10^{-34} \times 3 \times 10^{8}} = 1.12 \times 10^{19}$$

$$AQY = \frac{the\ number\ of\ evolved\ TEMPO\ molecules}{1.12 \times 10^{19}} = \frac{6.02 \times 10^{23} \times 5.37 \times 10^{-7}}{1.12 \times 10^{19}} = 2.8[illegible] \quad (6)$$

KIE(H/D) from AQY is KIE(H/D) = AQY(H)/AQY(D) =1.12

On pristine TiO_2 nanoparticles:

$H(CH_3OH)$:

$$N = \frac{E\lambda}{hc} = \frac{68 \times 1 \times 10^{-3} \times 90 \times 365 \times 10^{-9}}{6.626 \times 10^{-34} \times 3 \times 10^{8}} = 1.12 \times 10^{19}$$

$$AQY = \frac{the\ number\ of\ reduced\ TEMPO\ molecules}{1.12 \times 10^{19}} = \frac{6.02 \times 10^{23} \times 7.78 \times 10^{-7}}{1.12 \times 10^{19}} = 4.1[illegible] \quad (7)$$

D(CD₃OD):

$$N = \frac{E\lambda}{hc} = \frac{68 \times 1 \times 10^{-3} \times 90 \times 365 \times 10^{-9}}{6.626 \times 10^{-34} \times 3 \times 10^{8}} = 1.12 \times 10^{19}$$

$$AQY = \frac{the\ number\ of\ evolved\ TEMPO\ molecules}{1.12 \times 10^{19}} = \frac{6.02 \times 10^{23} \times 2.17 \times 10^{-7}}{1.12 \times 10^{19}} = 1.1[illegible] \quad (8)$$

KIE(H/D) from AQY is KIE(H/D) = AQY(H)/AQY(D) =3.60

KIE values of $^{t}Bu_3ArO\cdot$/TEMPO reduction calculated by comparing AQYs in H/D systems are consistent with our direct ESR experimental observations.
